# Plastome Structural Conservation and Evolution in the Clusioid Clade of Malpighiales

**DOI:** 10.1038/s41598-020-66024-7

**Published:** 2020-06-04

**Authors:** Dong-Min Jin, Jian-Jun Jin, Ting-Shuang Yi

**Affiliations:** 1 Germplasm Bank of Wild Species, Kunming Institute of Botany, Chinese Academy of Sciences, Kunming, China; 2 College of Life Sciences, University of Chinese Academy of Sciences, Beijing, China

**Keywords:** Molecular evolution, Plant evolution

## Abstract

The clusioid clade of Malpighiales is comprised of five families: Bonnetiaceae, Calophyllaceae, Clusiaceae, Hypericaceae and Podostemaceae. Recent studies have found the plastome structure of *Garcinia mangostana* L. from Clusiaceae was conserved, while plastomes of five riverweed species from Podostemaceae showed significant structural variations. The diversification pattern of plastome structure of the clusioid clade worth a thorough investigation. Here we determined five complete plastomes representing four families of the clusioid clade. Our results found that the plastomes of the early diverged three families (Clusiaceae, Bonnetiaceae and Calophyllaceae) in the clusioid clade are relatively conserved, while the plastomes of the other two families show significant variations. The Inverted Repeat (IR) regions of *Tristicha trifaria* and *Marathrum foeniculaceum* (Podostemaceae) are greatly reduced following the loss of the *ycf1* and *ycf2* genes. An inversion over 50 kb spanning from *trnK-UUU* to *rbcL* in the LSC region is shared by *Cratoxylum cochinchinense* (Hypericaceae), *T. trifaria* and *Ma. foeniculaceum* (Podostemaceae). The large inversed colinear block in Hypericaceae and Podostemaceae contains all the genes in the 50-kb inversed colinear block in a clade of Papilionoideae, with two extra genes (*trnK-UUU* and *matK*) at one end. Another endpoint of both inversions in the two clusioids families and Papilionoideae is located between *rbcL* and *accD*. This study greatly helped to clarify the plastome evolution in the clusioid clade.

## Introduction

Plastomes of heterotrophic plants are generally highly rearranged^1^, while plastomes of autotrophic angiosperms seem to be relatively conserved^2^. Most autotrophic angiosperm plastomes are characterized by a copy of Inverted Repeat (IR) regions, one Large Single Copy (LSC) region and one Small Single Copy (SSC) region, with the average size of 153 kb, generally include 101–118 unique genes that primarily participating in photosynthesis, transcription, and translation^3,4^. The advent of high-throughput sequencing has facilitated rapid progress in the field of comparative plastid genomics^5,6^.

Several distinct autotrophic angiosperms clades have substantial variations in plastome size and gene order. Large variation of plastome size is often associated with IR expansion or contraction^7^, but could also be influenced to some extent by gene and intron losses. The loss of two hypothetical open reading frames *ycf1* and *ycf2*, two largest plastid genes, could significantly reduce the plastome size^6^. Multiple independent losses of some plastid genes and introns have been reported^8,9^, some of these genes have transferred to the nucleus^9^. Successful gene transfers from the plastid to the nuclear genome during angiosperm evolution have been documented for *rpl22*, *rpl32* and *infA*^9,10^.

Inversions play an important role in plastid genome structural variations and have been fully characterized in a number of plastomes. Large inversions have been found in plastomes of many plant lineages, such as Onagraceae^11^, Asteraceae^12^, and Fabaceae^13^. In Fabaceae, multiple large inversions have been reported, including a 50-kb inversion shared by most Papilionoids except a few early-diverging clades, a 78-kb inversion in Phaseolinae of Phaseoleae, inversions of 23-kb, 24-kb, or 36-kb in the Genistoid clade, a 39-kb inversion in *Robinia* of Papilionoideae, and a 38-kb inversion in *Tylosema* of Cercidoideae^13–15^. Recent studies have found short Inverted Repeat (sIR) meditated flip-flop recombination event could induce large inversions^13,15,16^.

The clusioid clade (Malpighiales) contains five families (Bonnetiaceae, Calophyllaceae, Clusiaceae, Hypericaceae, and Podostemaceae) represented by 94 genera and ~1900 species^17^. Their distribution is nearly cosmopolitan, with the greatest species diversity in the tropics^18^. Species in this clade include large tropical rainforest trees, temperate and high-altitude tropical herbs and shrubs, and even aquatic plants (Podostemaceae) growing in swift-flowing rivers and streams^18^. Many species are economically important, such as tropical fruits including the mangosteen (*Garcinia mangostana* L.) and the mammey apple (*Mammea americana* L.), timber (*Calophyllum brasiliense* Cambess., *Mesua ferrea* L.), and medicine (*Hypericum perforatum* L.).

Previous studies found that the plastome of *Garcinia mangostana* L. from Clusiaceae was relatively conserved^19^, while plastomes of five riverweed species from Podostemaceae had highly variable structure^20^. Why closely related families have so diverged plastome structure? What is the plastome structural divergence pattern of this economically and ecologically important clade? The diversification pattern of plastome structure of the clusioid clade worth a further investigation. Here we determined five complete plastomes in the clusioid clade: *Bonnetia paniculata* Spruce ex Benth. (Bonnetiaceae), *Me. ferrea* (Calophyllaceae), *Cratoxylum cochinchinense* (Lour.) Blume. (Hypericaceae), *Tristicha trifaria* (Bory ex Willd.) Spreng. and *Marathrum foeniculaceum* Bonpl. (Podostemaceae). Comparison of the plastomes in this clade unveils significantly reduced IR regions in the plastomes of *T. trifaria* and *Ma. foeniculaceum* following the loss of *ycf1* and *ycf2*. A large inversion over 50 kb spanning from *trnK-UUU* to *rbcL* in the LSC region is shared by *C. cochinchinense*, *T. trifaria* and *Ma. foeniculaceum*.

## Results and Discussion

### Plastome sequencing and general characteristics

Raw reads were all obtained through whole-genome sequencing. Due to the differences in plant materials and experimental procedures, the average coverage depth of plastomes varies from 89 to 1823 (Tables 1, Table S1). All five new plastomes of the clusioid clade exhibit a typical quadripartite structure. The plastome size among the sampled clusioids species ranges from 130,967 bp in *T. trifaria* to 161,473 bp in *Me. ferrea*. The length of IR ranges from 19,916 bp in *Ma. foeniculaceum* to 27,614 bp in *Me. ferrea*. The GC content of *Ma. foeniculaceum* is slightly lower. The plastome size and IR length of the two species from the Podostemaceae are significantly smaller than those of the other three clusioids families.

**Table 1:** Statistics of five newly generated plastomes in the clusioid clade.

	*Bonnetia paniculata*	*Mesua ferrea*	*Cratoxylum cochinchinense*	*Tristicha trifaria*	Marathrum foeniculaceum
Family	Bonnetiaceae	Calophyllaceae	Hypericaceae	Podostemaceae	Podostemaceae
Size (bp)	156,782	161,473	156,953	130,967	131,600
Status	complete	complete	complete	complete	complete
Average base-coverage	89×	254×	382×	1823×	476×
Reads-used	30,000,000	30,000,000	30,000,000	20,570,154	19,714,250
IR size (bp)	27,309	27,614	26,086	19,599	19,916
Average read length (bp)	149	99	99	149	149
GC content	36.2%	36.4%	36.3%	36.3%	35.1%
Accession number	MK995182	MK995181	MK995180	MK995179	MK995178

### Phylogenetic relationships

**Figure 1:**
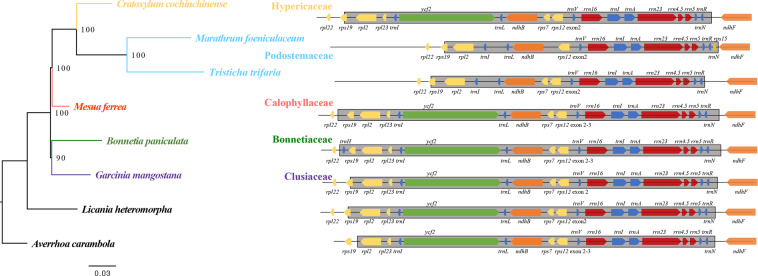
The plastid phylogeny of the clusioid clade. Maximum-likelihood (ML) tree inferred from the 82-gene (76 protein-coding and 4 rRNA genes) matrix. The number at each node indicates the ML bootstrap values. Species in the clusioid clade were color-coded according to family. A part of their plastome organization is shown on the right. The gray blocks indicate the IR regions. Gene lengths are not to scale. Gene arrow tips indicate the direction of transcription.

A maximum likelihood tree was constructed using an 82-gene matrix. The clusioid clade was strongly supported with a bootstrap value (BS) of 100%. Previous studies such as Ruhfel *et al*. (2011) using three plastid and one mitochondrial loci and Xi *et al*. (2012) using broad-range sampling plastome data also strongly supported the clusioid clade^17,21^. Our results are congruent with previous studies, which resolved a well-supported (Bonnetiaceae, Clusiaceae) clade as the early diverged lineage, and strongly supported Calophyllaceae being the sister to the strongly supported (Hypericaceae, Podostemaceae) clade^17,21,22^ (Fig. 1). There are also many morphological characteristics of species in this clade supporting these phylogenetic relationships. Though the position of the wholly aquatic Podostemaceae has been very difficult to be determined owing to their highly atypical morphology, the terrestrial members of this clade (i.e., Bonnetiaceae, Calophyllaceae, Clusiaceae, and Hypericaceae) have long been considered closely related^17,18,21^. Bonnetiaceae and Clusiaceae share staminal fascicles opposite the petals. Hypericaceae and Podostemaceae share tenuinucellate ovules^17^. Additionally, some members of Hypericaceae and Podostemaceae have papillate stigmas. Besides, Hypericaceae, Calophyllaceae, and some Podostemaceae share resin-containing glands or canals that are especially visible in the leaves^17^. The phylogeny of the clusioid clade provides a framework for understanding the evolutionary history of this morphologically and ecologically diverse clade.

### Plastome evolution

Plastomes structure of the early diverged three families (Clusiaceae, Bonnetiaceae and Calophyllaceae) are relatively conserved with only a few gene losses or pseudogenes. The *infA* gene and the second intron of *ycf3* are lost in the plastid genome of *G. mangostana, B. paniculata* and *Me. ferrea*. The *ndhK* gene is pseudogene due to the presence of an internal stop codon in *B. paniculata*. Other gene losses include the *rps16* gene in *B. paniculata* and the *rpl32* gene in *G. mangostana*. However, the other two families (Hypericaceae and Podostemaceae) show more considerable plastome structural variations. The plastomes of *C. cochinchinense, T. trifaria* and *Ma. foeniculaceum* have lost the *infA* and *rps16* genes, the second intron of the *clpP* gene, the second intron of the *ycf3* gene, and the intron of the *rps12* gene. The *rpl32* gene in *T. trifaria* and *Ma. foeniculaceum* and the *rps7* gene in *Ma. foeniculaceum* are pseudogenes due to the presence of premature stop codons. Additional gene losses in *T. trifaria* and *Ma. foeniculaceum* include plastid hypothetical ORFs (*ycf* genes), the *ycf1* and *ycf2* genes. As a result of the two *ycf* genes losses, the plastomes of the two Podostemaceae species are significantly smaller than the other four sequenced plastomes of the clusioid clade. IRs have expanded approximately 800 bp at the IR/SSC boundary in *Ma. foeniculaceum*, resulted in the relocation of the *rps15* gene from SSC to IR.

The two identical copies of IR provide a template for error correction when a mutation occurs in one of the copies, and hence likely suppress the substitution rate in the IR^3^. Previous studies have reported the increased substitution rate of genes relocated from IR into SC^23^, and the decreased substitution rate of genes relocated from SC into IR^24^. However, relocation of *ycf2* from IR into SC did not followed by an accelerated substitution rate, which has been explained by a recently occurred event in ginkgo evolution^25^. Studies in *Pelargonium* plastomes also found that expansion of IR does not result in decreased substitution rates of the relocated genes, suggesting the lineage- and locus-specific rate heterogeneity may have a larger effect that the IR on the substitution rate variation in plastid genes^3,24^. In our study, the relocated *rps15* gene didn’t show decreased substitution rate (LRT p-value: 0.21, df = 1, details in Table S2). Since the relocation of *rps15* did not accumulate significant mutations, we hypothesize that this relocation occurred recently or the *rps15* gene is simply too short for the substitution rate to be detected. Our study supplies another case that the gene relocated into IR does not show decreased substitution rates. Patterns of molecular evolution in the IR and SC regions differ, most notably by a reduced rate of nucleotide substitution in the IR compared to the SC region, but the evolutionary consequences may be more complex than previous suggested^3,24^.

The loss of *ycf1* and *ycf2* genes have been documented in the plastomes of Poaceae^26^, Geraniaceae^27^, and Ericaceae^28^. The functions of both *ycf* genes are still controversial. Studies in tobacco (*Nicotiana tabacum*) and green algae (*Chlamydomonas reinhardtii*) suggested the *ycf1* and *ycf2* genes should not be related to photosynthesis, but encode products that are essential for cell survival^29^. These two genes have been inferred to be involved in cell division, DNAs/mRNA binding, protein assembly and transport, etc^29^. One essential function of the *ycf1* and *ycf2* genes might be linked to expression, assembly, or function of the *accD* gene product^28,30^. Some Poaceae species have lost both *ycf* genes in addition to the *accD* gene^31^. Plants that have lost the *accD* gene have divergent *ycf1* and *ycf2* sequences^30^. The plastome of *T. trifaria* and *Ma. foeniculaceum*, which have lost *ycf1* and *ycf2*, have highly divergent *accD* sequences with only 51.8% and 51.1% identical sites, respectively, comparing with that of the early diverged three families (Clusiaceae, Bonnetiaceae and Calophyllaceae). Interestingly, the plastome of *C. cochinchinense*, which contains the two *ycf* genes, also has highly variable *accD* sequences with only 51.8% identical sites comparing with the firstly diverged three families. Further investigation is required to clarify the coevolution of *accD* and two *ycf* genes. Why plastomes of these taxa lost two *ycf* genes remains unclear, and they are also worth further explorations.

**Figure 2:**
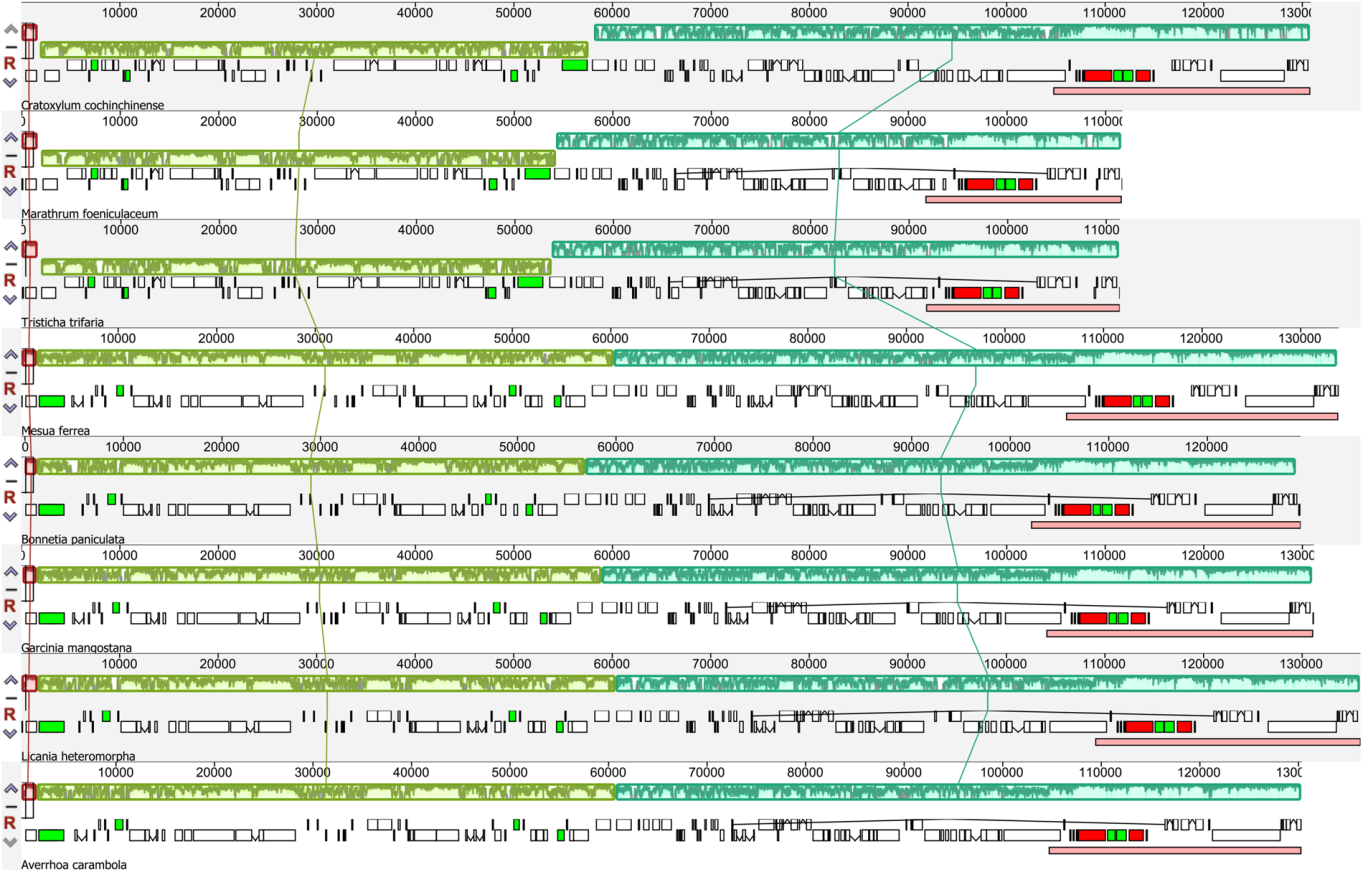
Synteny and rearrangements detected in eight plastomes using the progressiveMauve. Light green-colored regions represent the large inversion shared by the plastomes of *Cratoxylum cochinchinense* (Hypericaceae), *Tristicha trifaria* and *Marathrum foeniculaceum* (Podostemaceae).

Inversions have been fully characterized in a number of plastomes and represent an essential mechanism for plastome rearrangements^2^. A Large inversion spanning from *trnK-UUU* to *rbcL* in the LSC region is shared by three plastomes of Hypericaceae and Podostemaceae (56 kb and 52 kb respectively; Fig. 2). The inversions are about 4 kb shorter in *T. trifaria* and *Ma. foeniculaceum* than that in *C. cochinchinense*, mainly due to the loss of some intergenic sequences in the Podostemaceae plastomes. Parallel inversions utilizing the same endpoints in distantly related taxa are extremely rare^6^. Within Fabaceae, a 50-kb inversion occurs in most Papilionoideae except a few basal lineages^14,32^. Interestingly, the large inversion of Hypericaceae and Podostemaceae contains all the genes in the 50-kb inversion of Papilionoideae, with two extra genes (*trnK-UUU* and *matK*) at one breakpoint. Another breakpoint of this inversion is located between *rbcL* and *accD*, being identical to that of the 50-kb inversion of Papilionoideae. Earlier studies have demonstrated a strong correlation between repetitive sequences and the incidence of inversions. In several cases, dispersed repeats have been inferred to promote inversions through intramolecular recombination^2,15^. The distribution of repeats was found to be strongly associated with breakpoints in the rearranged plastomes of Geraniaceae^27^. Studies confirmed that a specific plastomic inversion of a 34-kb fragment in *Calocedrus macrolepis* was likely to be mediated by an 11-bp IR. A 36-kb inversion in *Lupinu*s and a 39-kb inversion in *Robinia* are probably mediated by a pair of 29-bp sIRs situated in the 3′-ends of two *trnS* genes^13^. No repeats have been found in boundary regions of the inversions in *T. trifaria* and *Ma. foeniculaceum*. While a pair of 76-bp sIRs are found at the breakpoints of the 56-kb inversion in *C. cochinchinense* (Fig. S1), which probably mediated this inversion.

## Methods

### Taxon sampling and DNA sequencing

The previously published plastome of *G. mangostana*^19^ (NC_036341) in this clade was included in comparative analyses. Illumina sequencing data of two species were obtained from the NCBI Sequence Read Archive (accession no. SRR7121482 and SRR7121944) representing two families in clusioids: *Me. ferrea* (Calophyllaceae) and *C. cochinchinense* (Hypericaceae). Three species were sampled to represent the other two families of this clade: *B. paniculata* (Bonnetiaceae), *T. trifaria* and *Ma. foeniculaceum* (Podostemaceae). Two representative species, *Licania heteromorpha* (NC_024062) from Malpighiales and *Averrhoa carambola* (NC_033350) from Oxalidales were included as outgroups.

Total genomic DNA of *B. paniculata*, *T. trifaria*, and *Ma. foeniculaceum* was isolated from specimens using the DNeasy Plant Mini Kit, then fragmented to construct short-insert (350 bp) library following manufacturer’s manual (Illumina). Paired-end sequencing was performed on Illumina HiSeq X TEN at Plant Germplasm and Genomics Center (Kunming Institute of Botany, Chinese Academy of Sciences). Details of sample collection are listed in Table S1.

### Genome assembly, annotation and analyses

The paired-end reads were filtered and assembled into complete plastome using GetOrganelle v1.6.1a^33–36^ under default settings, with kmers set dependent on the sequenced read length: -k 21,35,45,55,65,75,85,95,105,115,121 were used for 150-bp reads, while -k 21,45,65,85,89,91,95,99 were used for 100-bp reads. Final assembly graphs were checked in Bandage^37^. Two configurations of each plastome caused by the flip-flop recombination mediated by the IR were obtained, and one of the them was arbitrarily selected for downstream analysis since the plastome exists in two equimolar states^38^. All plastomes were initially annotated using PGA^39^ and GeSeq^40^, with annotated plastome from *Amborella trichopoda* (NC_005086) selected as the reference. For confirmation, all annotations were compared with the previously published plastome of *G. mangostana* and exon boundaries were manually adjusted in Geneious Prime^41^. All newly sequenced plastomes were deposited in GenBank under the accession nos. MK995178-MK995182. [Note to Reviewers: deposited sequences will be released immediately upon acceptance]

The 82 shared protein-coding and rRNA genes were extracted from the plastomes of eight species using “get_annotated_regions_from_gb.py” (https://github.com/Kinggerm/PersonalUtilities/)^42^, then aligned with prank v.140603^43^. Phylogenetic analysis was performed using maximum likelihood methods with 1000 bootstrap replicates on RAxML version 8.2.11^44^. We used codeml implemented in PAML^45^ to estimate nucleotide substitution rates of the *rps15* gene in *Ma. foeniculaceum* under the null model (1 dN/dS ratios for all branches) and alternative model (2 or more dN/dS ratios for branches). The codon frequencies were determined using F3 × 4 model. The 2-rate model was tested against the 1-rate model by LRT using chi2 in PAML. One copy of IR was removed from each plastome and the remaining genome sequences were aligned using the progressiveMauve algorithm in Mauve v2.3.1^46^. In order to identify and discard small or insignificant genome rearrangements, the minimum LCB weight was set as 1588. Repeats were identified using the Find Repeats implanted in Geneious Prime. The criteria used were set as follows: minimum repeat length: 30 bp, maximum mismatches: 3%, exclude repeats up to 10 bp longer than contained repeat and exclude contained repeats when longer repeats has frequency at least 3.

## Supplementary information

Supplementary Figure S1

Supplementary Table S1

Supplementary Table S2

## Data Availability

The complete plastome sequences of *Marathrum foeniculaceum*, *Tristicha trifaria*, *Cratoxylum cochinchinense*, *Mesua ferrea* and *Bonnetia paniculata* sequenced in this study has been submitted to GenBank database under accession numbers MK995178-MK995182.
