# Supplementary material for: Plastome Structural Conservation and Evolution in the Clusioid Clade of Malpighiales
Source: Sci Rep. 2020 Jun 4;10:9091. doi: 10.1038/s41598-020-66024-7 (PMC7272398; doi:10.1038/s41598-020-66024-7)
Supplement: Supplementary file 1 — Supplementary Figure S1 [file 41598_2020_66024_MOESM1_ESM.pdf]

Article title: Plastome Structural Conservation and Evolution in the Clusioid Clade of Malpighiales

Submitted to: *Scientific reports*

Author names: Dong-Min Jin, Jian-Jun Jin and Ting-Shuang Yi*

* Corresponding author

Germplasm Bank of Wild Species, Kunming Institute of Botany, Chinese Academy of Sciences, Kunming, China

tingshuangyi@mail.kib.ac.cn

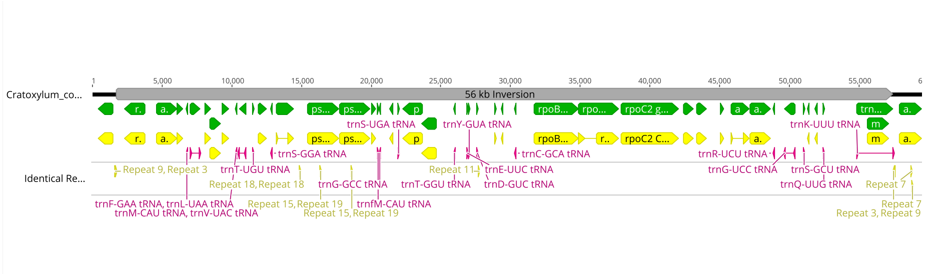

a. 56 kb inversion in *Cratoxylum cochinchinense* (Hypericaceae)

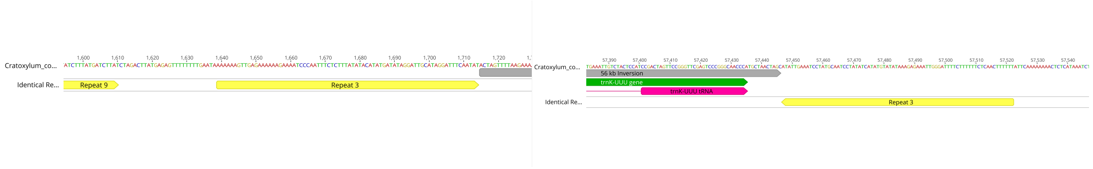

b. Forward 76 bp sIR

c. Reverse 76 bp sIR

Figure S1. The 56-kb inversion in *Cratoxylum cochinchinense* (Hypericaceae). A pair of 76-bp sIRs are found at the breakpoints of the 56-kb inversion in *C. cochinchinense*.
