# Supplementary material for: Plastome Structural Conservation and Evolution in the Clusioid Clade of Malpighiales
Source: Sci Rep. 2020 Jun 4;10:9091. doi: 10.1038/s41598-020-66024-7 (PMC7272398; doi:10.1038/s41598-020-66024-7)
Supplement: Supplementary file 2 — Supplementary Table S1 [file 41598_2020_66024_MOESM2_ESM.pdf]

Article title: Plastome Structural Conservation and Evolution in the Clusioid Clade of Malpighiales

Submitted to: *Scientific reports*

Author names: Dong-Min Jin, Jian-Jun Jin and Ting-Shuang Yi*

*Corresponding author

Germplasm Bank of Wild Species, Kunming Institute of Botany, Chinese Academy of Sciences, Kunming, China

tingshuangyi@mail.kib.ac.cn

Table S1 Details of sample collection

Species	Voucher, herbarium	Collected_by	Collection_date	Country	Identified_by	Lat_Lon
Bonnetia paniculata	David A. Neill - 15135, MO	David A. Neill, Thomas B. Croat & Wilson Quizhpe	4-Apr-06	Ecuador	D.A. Neill	3.58 S 78.44 W
Tristicha trifaria	W. D. Stevens - 36845, MO	W. D. Stevens	22-Aug-15	Nicaragua	W. D. Stevens	12.29 N 85.33 W
Marathrum foeniculaceum	W. D. Stevens - 32072, MO	W. D. Stevens & O.M. Montiel J	28-Oct-11	Nicaragua	W. D. Stevens	12.15 N 84.88 W
