# Supplementary material for: Plastome Structural Conservation and Evolution in the Clusioid Clade of Malpighiales
Source: Sci Rep. 2020 Jun 4;10:9091. doi: 10.1038/s41598-020-66024-7 (PMC7272398; doi:10.1038/s41598-020-66024-7)
Supplement: Supplementary file 3 — Supplementary Table S2 [file 41598_2020_66024_MOESM3_ESM.pdf]

Article title: Plastome Structural Conservation and Evolution in the Clusioid Clade of Malpighiales

Submitted to: *Scientific reports*

Author names: Dong-Min Jin, Jian-Jun Jin and Ting-Shuang Yi*

*Corresponding author

Germplasm Bank of Wild Species, Kunming Institute of Botany, Chinese Academy of Sciences, Kunming, China

tingshuangyi@mail.kib.ac.cn

Table S2. Summary of the log-likelihood test (LRT) results using PAML

locus	lnLH0	lnLHA	lnLHA-lnLH0	*P* value (P<0.05)
rps15	-1018.2789	-1017.496171	0.782697	0.21
